# ‘Becoming a Physician’–medical students get acquainted with disadvantaged populations, and practise sensitive and effective communication

**DOI:** 10.1007/s40037-015-0222-8

**Published:** 2015-10-19

**Authors:** Arieh Riskin, Nogah C. Kerem, Riki Van-Raalte, Michael Kaffman, Gila Yakov, Dror Aizenbud, Ayelet Ben-Barak, Yael Shachor-Meyouhas, Ruth Edery, Yael Shabtai Musih, Shlomi Sagi, Orly Eidelman, Avi Rotschild

**Affiliations:** 1 Ruth & Bruce Rappaport Faculty of Medicine, Technion, Israel, Institute of Technology, Haifa, Israel; 2 Department of Neonatology, Bnai Zion Medical Center, 47 Golomb Street, PO Box 4940, 31048 Haifa, Israel

**Keywords:** Medical students, Exposure, Community, Communication, Disadvantaged

## Abstract

**Background:**

The three-year pre-medical programme ‘Becoming a Physician’ focuses on different aspects of medical professionalism. Objectives are to increase awareness and sensitivity to disadvantaged populations, and practise sensitive effective communication skills.

**Methods:**

The curriculum includes: (1) Visits to treatment centres for people with special needs, mental illnesses, substance abuse issues, physically or sexually abused, and prisoners. Students tour the facility, hold discussions with residents, and discuss ethical professional interrelations to the medical world. Students then write ‘reflective diaries’ summarizing their thoughts and emotions. (2) Participation in a communication course that focuses on learning by practising patient-oriented communication. Qualitative data were collected from three sources: reflective diaries, studentsʼ course evaluations, and interviews with the studentsʼ tutors.

**Results:**

Data indicated that the students were very satisfied with the programme. They indicated an increase in awareness of the special needs of diverse populations, and in the sense of efficacy for conducting interviews tailored to patients' needs. Tutors reported a sense of ‘personal growth’ following their role as mentors.

**Reflections:**

Interactions of medical students with diverse populations, when accompanied by appropriate feedback mechanisms and strengthening of communication skills, can improve awareness and sensitivity to patients' special needs. This could help students become more sensitive and thoughtful physicians.

## Background

The concern for sustaining compassion and communication skills among students of medical schools who will be our future physicians has been extensively discussed yet we are far from good solutions [1]. Over the last 20 years medical students of the Technion participated in a unique three-year programme *Becoming a Physician—Exposure to the Medical World* during their pre-medical years. The students are divided into small heterogeneous groups (8–10 students). Meetings are held every other week (approximately 18 meetings each year for three years). To reach maximal effectiveness, meetings were designed based on Gagné’s nine events of instruction [2, 3], including debriefing at the end of each session and personal feedback between sessions. Each group is led by a tutor. The tutors are physicians, representing a wide range of ages, specialities and levels of training, who are carefully selected so that they can serve as professional role models. Being chosen to serve as a mentor is perceived as prestigious in our faculty, a token of appreciation for one's personal, professional and academic qualities. The content of the course is appealing to most physicians and a way to enrich their daily routine, so many are happy to volunteer.

The programme objective is to expose young students to different psycho-social aspects of the medical profession during the years devoted to pre-med scientific studies so they would be better prepared for their clinical years. This includes: different working environments; multidisciplinary professionals; patients with diverse sociocultural backgrounds; challenging ethical issues; different aspects of the ‘medical life’; and impacts of professional life on one’s personal life. The programme also aims to teach and practise patient-centred communication, using Gagné’s nine steps of instruction [2, 3]. Students share their feelings, thoughts and experiences in a formal manner at closing discussions that are held at the end of each meeting, as well as informally during their after-school get-together. The programme also gives the students a ‘second chance’ to examine their choice to become physicians and their personal compatibility for medical life. The interactive nature allows tutors to identify students who may benefit from emotional support or professional consultation regarding their choice of a medical career.

The objective of this Show and Tell contribution is to introduce our unique pre-medical educational programme, present some –preliminary– qualitative evaluations, and discuss our reflections on the programme.

## Course curriculum

Different aspects of the physician’s work are stressed during the programme. In the first year students are introduced to the hospital setting—inpatients, different departments and professions. In the second year students reach out to the community, meet disadvantaged populations, learn and practise effective and sensitive patient-centred communication. In the third year multi-cultural aspects of medicine are presented, and the students are challenged with complex ethical dilemmas using insights they have sustained from their two previous years. Second year programme curriculum includes:

1. Meeting disadvantaged populations. The goal is to familiarize students with populations that need special attention and care; deal with dogmatic beliefs, prejudice and emotions that arise from meeting the unfamiliar, including: elderly people in geriatric hospitals or nursing homes; children and adults with special needs (mental or physical handicaps); people with mental illnesses in hospitals or in community hostels; battered women in shelters, and sexually abused women; people with drug or alcohol addiction; and prisoners in jail. A typical meeting in a community care centre includes an introduction about the goals and special services the facility provides to their unique population; meetings between students and the facility’s residents; and a final gathering with their tutor for ventilating emotions, sharing thoughts and discussing professional and ethical linkages to the medical world.
2. Improving communication skills. The goal is to enhance communication skills emphasizing sociocultural sensitivity. The methods include a theoretical session on communication methods, based on the key tasks in communication, as described by Maguire & Pitceathly [4]; a practice session using role play; practising patient-physician communication in primary clinics; and implementation of communication skills learnt during each visit to a community centre.

## Course evaluation

We use three qualitative evaluation methods: (1) Studentsʼ feedback questionnaires (150 feedback questionnaires reviewed). (2) Oral feedback sessions with the tutors (8 annual sessions with 10–15 tutors each were reviewed). (3) Studentsʼ ‘reflective diaries’. Following each meeting students wrote diaries about their reflections following the visit. The tutors send written feedbacks (36 annual diaries of 14 meetings each were studied).

Overall the programme ranked highly in studentsʼ satisfaction questionnaires, mostly because they felt that their voice was heard, that the discussions within the group were respectful and non-judgmental, and that they could freely express their emotions. Many said that it was the most significant programme during their pre-clinical studies. Students were satisfied with meeting disadvantaged populations. Many asked: ‘*How come I was never introduced to such populations before?*’ Students and tutors reported an increase in their awareness and sensitivity to the special needs of different populations, and felt that their interviewing skills had significantly improved. Selected quotes describing students and tutors impressions are presented in Tab. 1. Some students who had personally experienced a health/social issue affecting the populations they met (e.g. having a sib with special needs) reported that they finally had a chance to openly and intimately discuss their feelings with fellows, and that their tutors provided emotional support.

**Tab. 1:** Selected quotes describing students and tutors’ impressions

After a visit to a hostel for battered women a student wrote: *‘Even though the hostel is adjacent to my home, I had no idea who lived there, and why the doors were always locked. The women, who always entered the house very quickly, were transparent to me. I had no idea what burdens they carried on their shoulders… It’s not that I didn’t want to know more, I simply had no way or opportunity to get closer’*
Following a visit to a day-care facility for adults with severe mental retardation another student wrote: *‘My attitude to people suffering from mental retardation has completely changed’; ‘I was surprised at how much every activity taking place in the day-care was oriented to allow free choice for the residents, and to respect their free will, even of the most profoundly mentally challenged people’; ‘Now I see “them” as human beings, who can be sad or happy, who have their own desires, which we need to respect’*
A department head, who was a tutor said: *‘I was not only exposing my students to the medical world, I felt as if I was actually exposing myself. It touched me on many different levels, it made me think … I’ve never thought that at my age and in my professional status it could touch me this way. I can say that I’ve experienced personal growth, and to say that on the verge of my retirement is not trivial at all’*
The last quote is from a house officer during his residency in medicine who participated in the programme as a student, and made a point to write to us after a night call in the ER: *‘I saw the prisoner in the emergency department, handcuffed to his bed. Every doctor secretly moved down his chart to the bottom of the pile, in order to avoid the need to deal with him… I suddenly recalled my visit to a jail when I was a second year student in the ‘Becoming a Physician’ programme, and remembered a conversation I had with a young prisoner who suffered from Crohn’s disease. He told me about his negative experiences from our hospital’s emergency department, and I decided that I was not going to let this happen again. I took a deep breath, picked the prisoner’s chart, asked his guards to step back, and treated him as any other patient. His thanks at the end of my visit meant the world to me’*

## Discussion

In the past academic medical education was strictly separated between ‘pre-clinical’ (theoretical basic sciences) and ‘clinical’ (rotations in hospital departments) years [5]. The trend nowadays is to incorporate some elements of clinical exposure in a spiral fashion along all the years, including early pre-clinical years. Dornan et al. showed that such early clinical experience eased students’ introduction into the clinical environment, improved their motivation, strengthened their confidence in approaching patients, increased their awareness of themselves and others, and clarified the role of medical profession within the social and behavioural context of health care organizations [6]. Early experience in the community helped students acquire basic communication and clinical skills that were invaluable to them. Community exposure enabled students to improve their affective skills, such as empathy; increased their self-awareness; helped them feel more content with their choice to practise medicine; and decreased their anxiety associated with meeting patients. Studentsʼ exposure to the community early in the course of their medical studies also contributed to their tutors, health care providers in the community and patients [7].

Teaching communication skills as part of the curriculum of medical studies is gaining popularity and is very successful [4, 8–10]. Yedidia et al. [11] have shown that developing a comprehensive educational syllabus in the field of communication skills, and implementing it from the very early stages, improved studentsʼ communication abilities, and improved their skills in building inter-personal relations—skills that are important for the success of treatment and for patient satisfaction [11–13]. Exercise, feedback and reflection are integral parts of such courses. The use of reflection techniques as an educational and feedback tool encourages processing, introspection and critical thinking on all actions and experiences of the individual and team, thus encouraging learning and improvements [14, 15].

Our programme holds many of these educational goals: exposing young students to different aspects of the medical profession; enhancing their sensitivity to special needs of disadvantaged populations; improving their communication skills, and their ability to reflect on their experiences.

We believe that this programme held positive effects on increasing medical studentsʼ awareness and sensitivity to the special needs of disadvantaged populations. This was mainly achieved by means of personal unmediated meetings with a diversity of people, accompanied by a communication skills’ course and appropriate feedback mechanisms. The sensitivity of our students developed concomitantly with their sense of comfort to conduct patient-centred interviews.

The main limitation is that the evaluations are based on self-report questionnaires and diaries, and unfortunately the relation between self-reports and what actually happens in real life is not necessarily very strong. Furthermore, the findings of the evaluation are, due to its qualitative nature, subjected to our interpretation.

Although all students studied at one faculty, the programme involved 7 hospitals and over 20 community facilities, thus students met different patients and had different experiences, which is true for any clinical rotation.

## Conclusion

Based on our impression, we suggest that exposure of young medical students to disadvantaged populations in the community, when accompanied by appropriate feedback mechanisms, and with measures to strengthen communication skills, improves students’ understanding of special needs of different populations. The communication skills acquired included: patient-centred communication, listening, using open questions and loads of empathy. This should enhance the ability of our students to become more sensitive and attentive physicians to their patients.

Further studies should be carried out in order to correlate the subjective experiences of our students and mentors to other outcomes such as objectively measured communication skills with a diverse group of patients.
